# Green synthesis of Fe_2_O_3_-MnO_2_ nano-hybrids on pumice for complete degradation of pharmaceutical pollutants

**DOI:** 10.1371/journal.pone.0334324

**Published:** 2025-10-10

**Authors:** Abdulrazzaq Hammal, Ahmad Sulaiman

**Affiliations:** 1 Department of Basic Sciences, College of Electrical and Electronic Engineering, University of Aleppo, Aleppo, Syria; 2 Department of Chemistry, College of Science, University of Tartous, Tartous, Syria; Indian Institute of Technology Delhi, INDIA

## Abstract

Pharmaceutical contaminants in wastewater pose critical environmental and public health risks due to their persistent nature and resistance to conventional treatment. To address this challenge, we developed an innovative, green-synthesized Fe_**2**_O_3_-MnO_**2**_ nano-hybrid catalyst supported on acid-activated Syrian pumice. The catalyst was prepared via an eco-friendly hydrothermal method using Laurus nobilis leaf extract as a bio-reducing agent, emphasizing sustainability. Structural characterization revealed significant enhancement in surface properties, with the modified catalyst exhibiting a high surface area (214.7 ± 3.5 m^2^/g) and optimized pore architecture (0.36 cm^3^/g volume, 6.7 nm average diameter) featuring 80% mesopores and 20% micropores. Under mild conditions (pH 7, 25°C, 0.5 g/L catalyst dose, 10 mM peroxymonosulfate), the system achieved 92.3% COD and 93.5% BOD_5_ removal within 3 hours for wastewater laden with the beta-lactam antibiotic amoxicillin (50 mg/L). LC-MS/MS analysis confirmed the complete degradation of the target pollutants, with no toxic intermediate byproducts detected. The catalyst exhibited exceptional stability, retaining >86% efficiency after five reuse cycles, with minimal metal leaching (Fe/Mn < 0.3 mg/L, within WHO limits). In continuous-flow mode, it maintained 89.6% COD and 90.4% BOD_5_ removal, demonstrating scalability. This study bridges nanotechnology and circular economy principles by valorizing locally abundant volcanic pumice (a natural waste) through an eco-friendly synthesis route, presenting a scalable and industrially viable solution for pharmaceutical wastewater treatment.

## 1. Introduction

Pharmaceutical wastewater and pharmaceutical and personal care products (PPCPs) waste represent a significant environmental and technical challenge due to their complex chemical composition and content of toxic, persistent organic compounds such as antibiotics, steroids, and hormones, which exhibit toxic effects even at low concentrations [1–3]. Conventional wastewater treatment plants face major difficulties in removing these emerging contaminants, as they primarily rely on traditional biological processes targeting biodegradable organic matter, limiting their removal efficiency of pharmaceutical compounds to only 20–60% [4,5]. Traditional treatment technologies such as advanced oxidation processes (AOPs) also face challenges related to catalyst costs and post-treatment separation difficulties, especially when using free nanoparticles, in addition to membrane pore clogging issues, reduced long-term efficiency, and the potential formation of toxic byproducts [6–8].

To address these challenges, hybrid technologies integrating multiple treatment processes have emerged to achieve a synergistic effect in contaminant removal. For example, combining activated carbon adsorption with nanofiltration membranes can increase removal efficiency to 99% for some compounds while reducing membrane fouling issues and extending sorbent lifespan [9–11].

Systems combining photocatalysis and biological processes have shown high effectiveness in removing antibiotics such as tetracycline and sulfamethoxazole, achieving up to 95% removal in short time periods, while advanced oxidation systems coupled with membrane filtration achieved high efficiency in complete degradation of hormones and steroids [12,13].

Pumice has been selected as a natural, low-cost, highly porous catalyst support for effective nanoparticle distribution due to its chemical stability in aqueous environments, making it suitable for long-term industrial applications compared to other materials such as zeolite or silica that require expensive processing [14].

For instance, Yang et al. recently demonstrated the effectiveness of a magnetic MnFe_**2**_O_4_/pumice composite for peroxodisulfate activation and levofloxacin degradation. However, their synthesis relied on conventional chemical co-precipitation methods [14]. In contrast, our study advances the field by developing a bimetallic Fe-Mn oxide catalyst on pumice via a green synthesis route, eliminating the use of hazardous chemical reducing agents.

The proposed system relies on the synergy between iron (Fe_**2**_O_3_) and manganese (MnO_**2**_) components, where the multiple oxidation states of manganese (Mn^3+^/Mn^4+^) activate peroxymonosulfate (PMS), while iron (Fe^2+^/Fe^3+^) generates hydroxyl (-OH) and sulfate (SO_4_- ⁻) radicals to ensure multi-pathway attacks on pharmaceutical pollutants [14,15]. Recent studies have explored bimetallic Fe-Mn oxides for PMS activation due to their synergistic redox coupling (Fe^3+^/Fe^2+^ and Mn^4+^/Mn^3+^), which enhances radical generation [15]. Building on this foundational work by Shi et al. [15], which highlighted the enhanced electron transfer in a Fe_3_O_4_-MnO_**2**_ nanocomposite, our work extends the concept by immobilizing a similar synergistic Fe-Mn oxide system onto a low-cost, natural pumice support and employing a fully green synthesis methodology, thus addressing the sustainability and cost limitations often associated with nanomaterial production. For instance, Shi et al. reported a Fe_3_O_4_-MnO_**2**_ nanocomposite for enhanced pollutant degradation. However, many of these systems rely on chemical synthesis routes, expensive supports, or complex electrochemical setups [15,16]. This study differentiates itself by developing a green-synthesized Fe_**2**_O_3_-MnO_**2**_ nano-hybrid supported on low-cost, naturally abundant pumice, leveraging a bio-reducing agent (Laurus nobilis extract) to promote sustainability and reduce environmental footprint.

This approach aligns with the growing trend of using bio-derived materials in catalysis, such as the use of chitosan-coated catalysts for persulfate activation [17], but distinguishes itself by utilizing an entirely plant-based extract for reduction and a mineral-based (pumice) support, further enhancing the environmental credentials and reducing the overall cost of the catalyst [17,18].

Addressing these economic and sustainability challenges requires the development of innovative catalytic systems that are both highly efficient and environmentally benign. This study presents an advanced hybrid catalytic system based on nanoscale iron-manganese oxides supported on pumice for effective pharmaceutical wastewater treatment. The system features a unique design that combines the exceptional structural properties of pumice with the electronic synergy between iron and manganese components, enabling highly efficient treatment of a wide range of complex pharmaceutical contaminants.

The system’s performance relies on the integrative interaction between three key components. The pumice substrate provides a hierarchical porous structure that facilitates optimal nanoparticle distribution and efficient contaminant transport to active sites. The iron oxide (α-Fe_**2**_O_3_) component contributes visible-light responsiveness and excellent reactive oxygen species generation capability, while manganese oxide (MnO_**2**_) complements this mechanism through its variable oxidation states, enabling peroxymonosulfate activation and resistant chemical bond cleavage.

The catalytic mechanism follows an integrated sequence: initial contaminant concentration on the pumice’s porous surface, followed by progressive oxidation through iron oxide-generated reactive oxygen species, and final degradation completion via manganese oxide, ultimately achieving complete mineralization of contaminants into harmless byproducts. The synergistic interaction between these components significantly enhances system efficiency while maintaining high stability over multiple usage cycles.

This study represents a qualitative advancement in wastewater treatment technology, offering a comprehensive solution to challenges posed by diverse pharmaceutical contaminants with varying chemical stability levels. The system combines scientific and practical advantages, including high efficiency, low energy consumption, and large-scale applicability, making it a promising candidate for industrial applications in pharmaceutical wastewater treatment.

## 2. Materials and methods

### 2.1. Preparation of acid-activated pumice support

Syrian pumice was selected as a catalyst support due to its local availability, low cost, high porosity, and chemical stability in aqueous environments, making it a superior and sustainable alternative to synthetic supports like zeolite or silica which often require expensive processing [14]. The pumice substrate was prepared from natural Syrian volcanic rock with an initial particle size range of 1–3 mm. Prior to processing, the raw pumice was chemically characterized using X-ray fluorescence (XRF) spectroscopy (Bruker S8 TIGER, wavelength-dispersive XRF spectrometer) equipped with a Rhodium anode X-ray tube (4 kW max power). Measurements were performed under vacuum conditions with a voltage of 60 kV and current of 150 mA, utilizing a LiF(220) analyzer crystal for elemental quantification. Primary processing involved mechanical size reduction using an agate mortar followed by sieving to obtain 300–500μm fractions. The sieved material underwent rigorous cleaning through sequential ultrasonication (40 kHz, 30 min) in deionized water until the supernatant exhibited negligible turbidity, as confirmed by spectrophotometric analysis at 600 nm.

Surface activation was achieved through controlled acid treatment using 2M hydrochloric acid (prepared from 37% ACS-grade HCl) at a solid-to-liquid ratio of 1:5 (w/v). The acid leaching process was conducted under reflux conditions at 70 ± 0.5°C with constant mechanical stirring (300 rpm) for precisely 4 hours to ensure uniform surface modification while preserving the structural integrity of the pumice matrix. Following activation, the material was subjected to exhaustive washing with deionized water until the effluent reached pH 6.8 ± 0.2, indicating complete removal of residual acid and soluble species.

Purified pumice was separated by centrifugation (4000 rpm, 5 min) and dried to constant weight at 105 ± 2°C in a convection oven with forced air circulation. Final moisture content was maintained below 0.5% as determined by thermogravimetric analysis. For storage, the activated pumice was kept in amber glass containers with silica gel desiccant to prevent surface hydration and atmospheric contamination.

### 2.2. Green synthesis of Fe_2_O_3_-MnO_2_ nano on activated pumice

The process employed high-purity reagents from Sigma-Aldrich, including iron(III) chloride hexahydrate (FeCl_3_·6H_**2**_O, purity ≥99%), potassium permanganate (KMnO_4_, purity ≥99.5%), and sodium hydroxide (NaOH, purity ≥98%).

The synthesis began with the preparation of the green reducing agent from *Laurus nobilis* (bay) leaves, which was selected for its high content of polyphenols and flavonoids, acting as effective reducing and capping agents for metal oxide nanoparticle synthesis, Laurus nobilis leaves collected from Syrian coastal regions. The leaves underwent sequential washing with deionized water and controlled drying (28 ± 2°C, 45 ± 5% relative humidity) for 72 hours. After grinding, a 12% (w/v) aqueous extract was prepared by steeping 12 g of plant powder in 100 mL deionized water at 70°C for 45 minutes with constant stirring (350 rpm), followed by filtration through 0.45 and 0.22 μm membranes (Whatman).

For the nano-hybrid formation, Stoichiometric amounts of metal precursors (2.703 g FeCl_3_·6H_**2**_O and 1.581 g KMnO_4_) were dissolved to prepare 0.1M solutions, aiming for a 1:1 molar ratio of Fe:Mn, which has been reported in literature to optimize the synergistic redox interaction between the two metals for PMS activation [15].

These solutions were combined in a three-neck reactor, with pH adjusted to 9.0 ± 0.2 using 1M NaOH. The bay leaf extract was introduced dropwise (0.4 mL/min) to initiate the reduction process, followed by gradual addition of 5g activated pumice support. The mixture underwent hydrothermal treatment at 80°C for 6 hours in a Teflon-lined autoclave, yielding the final nanocomposite after washing and drying.

The X-ray diffraction (XRD) pattern of the activated pumice and modified pumice were recorded using a Bruker D8 Advance diffractometer equipped with a Cu-Kα radiation source (λ = 1.5406 Å). Measurements were performed in the 2θ range of 5° to 50°, with a scan rate of 0.02°/s, at an operating voltage of 40 kV and a current of 40 mA. The data collection time was set to 1 second per step.

### 2.3. Brunauer–Emmett–Teller (BET) surface area analysis methodology

Surface properties were evaluated through N_**2**_ physisorption at 77 K using a Gemini 2375 volumetric analyzer (Micromeritics, USA). The specific surface area was calculated using the Brunauer–Emmett–Teller (BET) method from the adsorption data in the relative pressure (P/P_0_) range of 0.05–0.30. The total pore volume was estimated from the amount adsorbed at a P/P_0_ of ~0.95. The pore size distribution was derived from the adsorption branch of the isotherm using the Barrett–Joyner–Halenda (BJH) model [19,20].

### 2.4. Treatment of pharmaceutical wastewater and analytical procedures

Pharmaceutical wastewater was selected for this study because it contains a mixture of beta-lactam antibiotics, which are among the most prevalent and hazardous pharmaceutical pollutants due to their resistance to biological degradation and their potential to promote antibiotic-resistant bacterial strains when released into the environment. A comprehensive qualitative and quantitative analysis of the collected effluent using LC-MS/MS confirmed amoxicillin as the dominant contaminant at an initial concentration of ~10 mg/L, alongside other trace pharmaceutical compounds. Therefore, amoxicillin was selected as the representative model pollutant for this study. These characteristics make its treatment an urgent environmental and public health priority.

Samples were collected from the effluent of a local pharmaceutical wastewater treatment unit following standard protocols (EPA Guidance Manual for Sampling and ISO 5667−3) No specific permits were required for the collection of the wastewater samples used in this study, as they were obtained from the effluent of a local pharmaceutical treatment unit through a collaborative agreement. for research purposes. They were transported in amber glass containers and stored at 4°C to prevent any degradation or chemical changes. Prior to experimentation, the samples underwent precise filtration using 0.45μm membrane filters to remove suspended solids, followed by preliminary analyses including Chemical Oxygen Demand (COD) and Biochemical Oxygen Demand (BOD_5_), COD measurements were performed using the closed reflux colorimetric method according to Standard Method 5220 D with aHach DR3900 spectrophotometer. BOD_5_ measurements were carried out using the manometric method with WTW OxiTop® IS 6 measuring systems, following Standard Method 5210

A comprehensive qualitative and quantitative analysis of the pharmaceutical effluent was performed using liquid chromatography-tandem mass spectrometry (LC-MS/MS), The analysis was performed using an Agilent 1260 Infinity II liquid chromatography system coupled to an Agilent 6470 triple quadrupole mass spectrometer (Agilent Technologies, USA). The separation was conducted using a C18 column (2.1 mm internal diameter, 100 mm length, 1.8 µm particle size). The mobile phase consisted of deionized water with 0.1% formic acid (A) and methanol with 0.1% formic acid (B), operated in a gradient mode. The flow rate was maintained at 0.3 mL/min with an injection volume of 10 μL. The mass spectrometer operated in the positive electrospray ionization (ESI+) mode, with multiple reaction monitoring (MRM) transitions selected for each analyte. Source temperature was set at 350°C, the source voltage at 4000 V, and a nitrogen flow of 10 L/min was used as the nebulizer gas.

All catalytic degradation experiments in this study were conducted under dark conditions (in the absence of any external light source) to isolate and confirm the chemical catalysis pathway of the oxidant (PMS). These meticulous preparation procedures ensured the acquisition of homogeneous and reliable samples for all subsequent experiments, which investigated the effects of various factors such as pH, catalyst concentration, temperature, and treatment time. Throughout these studies, the fundamental characteristics of the samples remained consistent while the investigated variables were systematically altered to determine optimal treatment conditions.

The pH values of pollutant solutions with a concentration of 50 mg/L were adjusted to the range of 3–11 using 0.1 M HCl and NaOH solutions. A hybrid catalyst (0.5 g/L) and 10 mM PMS oxidant were added to each sample. COD and BOD_5_ measurements were taken every 30 minutes for 180 minutes using Hach DR3900 and WTW OxiTop instruments following standard methods.

Different concentrations of the hybrid catalyst (0.1–1.0 g/L) were tested while maintaining pH at 7 and PMS concentration at 10 mM. COD and BOD_5_ concentrations were monitored every 30 minutes for 3 hours under constant temperature conditions (25 ± 1°C).

The effect of temperature (25–80°C) on treatment efficiency was studied while maintaining pH at 7 and catalyst concentration at 0.5 g/L. A precise thermostatic water bath was used to control temperature, and COD and BOD_5_ were measured every 30 minutes.

The temporal evolution of the treatment process was studied under the optimal conditions identified above (pH 7, 0.5 g/L catalyst concentration, 25 ± 1°C). These conditions were chosen based on the prior parametric studies to ensure that the kinetic data reflect the system’s performance under optimized operational parameters.

Samples were collected at regular intervals (15, 30, 60, 90, 120, and 180 minutes) following the addition of 10 mM PMS oxidant. COD and BOD_5_ measurements were performed at each time point using Hach DR3900 and WTW OxiTop instruments following standard methods.

### 2.5. Stability and reusability of the hybrid catalyst

The performance of the hybrid catalyst (Fe_**2**_O_3_-MnO_**2**_/pumice) was evaluated over five consecutive cycles under optimal conditions (pH 7, concentration 0.5 g/L, temperature 25°C). After each cycle, the catalyst was separated by centrifugation (4000 rpm for 5 minutes), washed with a 0.1 M sodium hydroxide solution to remove organic residues, and dried at 105°C for two hours. Metal leaching (iron and manganese) was measured using inductively coupled plasma optical emission spectrometry (ICP-OES), the surface area and pore volume were measured using the BET method before and after each cycle to monitor physical changes.

### 2.6. Design and configuration of the treatment reactor

The integrated treatment system developed in this study synergistically combines physical filtration, heterogeneous catalysis, and advanced oxidation processes within a single continuous-flow reactor setup. Using pumice as a natural porous support, iron-manganese nano-hybrids catalyze the activation of peroxymonosulfate (PMS) to generate reactive oxygen species (ROS), effectively degrading persistent pharmaceutical contaminants in wastewater. This approach bridges the efficiency gap between conventional biological treatments—often insufficient for micropollutant removal—and energy-intensive advanced oxidation techniques by offering a scalable, ecofriendly, and cost-effective alternative suitable for industrial applications

**Fig 1:**
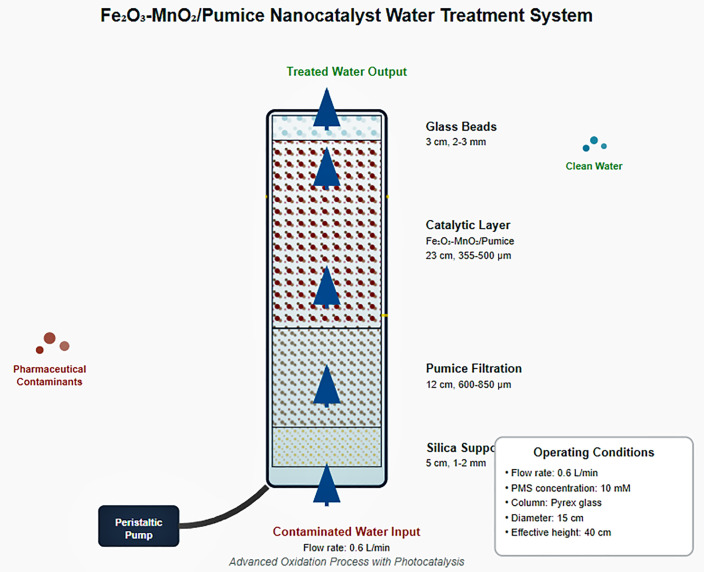
Structural design of the integrated treatment unit based on Fe_2_O_3_-MnO_2_/pumice.

The system was constructed using a cylindrical glass (Pyrex) column (15 cm internal diameter, 40 cm effective height) equipped with a precision flow control system operated by a Masterflex L/S peristaltic pump (model 7523−40) and Tygon® LFL 180 tubing (8 mm internal diameter). The column consists of four consecutive layers: a supporting silica layer (5 cm, 1–2 mm), a pumice filtration layer (12 cm, 600–850 μm), a catalytic layer (23 cm, 355–500 μm), and a distribution layer of glass beads (3 cm, 2–3 mm). Operational results at a flow rate of 0.6 L/min and a PMS concentration of 10 mM Fig 1. Each experiment was performed at least in triplicate (n = 3), and results are presented as mean ± standard deviation

### 2.7. Toxicity assessment bioassay

The ecotoxicological impact of the wastewater before and after treatment was evaluated using the luminescence inhibition assay with the marine bacterium *Vibrio fischeri* (according to the standard ISO 11348−3 protocol).

Briefly, lyophilized bacteria (e.g., from a kit like “BioTox^TM^”) were rehydrated with the provided reconstitution solution. Serial dilutions of both the raw wastewater and the treated effluent were prepared using 2% NaCl solution to maintain osmotic pressure for the marine bacteria. Each sample dilution was incubated with the bacterial suspension at 15°C for 30 minutes.

The luminescence of the samples was then measured using a luminometer (e.g., a Microtox® Model 500 analyzer or similar). The inhibition of luminescence (%) for each sample was calculated by comparing its light output to that of a control (2% NaCl solution instead of the sample). The EC_50_ value (the effective concentration that causes 50% inhibition of luminescence) was determined from the dose-response curve using appropriate software or statistical analysis. All tests were performed in triplicate (n = 3), and results are presented as mean ± standard deviation.

## 3. Results and discussion

### 3.1. Fundamental characteristics of Syrian pumice

X-ray fluorescence (XRF) analysis revealed the Syrian pumice to be a high-silica volcanic material, with SiO_**2**_ (72.5 ± 0.8 wt.%) and Al_**2**_O_3_ (13.2 ± 0.5 wt.%) constituting 85.7% of its bulk composition. This chemical signature classifies the material as a rhyolitic pumice according to the TAS (Total Alkali-Silica) classification scheme [21], consistent with its formation from viscous, silica-rich magmas during explosive volcanic eruptions Table 1

**Table 1:** Chemical Composition Raw Pumice.

Oxide	Composition (wt.%)
SiO_**2**_	72.5 ± 0.8
Al_**2**_O_3_	13.2 ± 0.5
K_**2**_O	4.8 ± 0.3
Na_**2**_O	3.6 ± 0.2
CaO	2.1 ± 0.1
Fe_**2**_O_3_	1.9 ± 0.1
MgO	0.7 ± 0.05
TiO_**2**_	0.5 ± 0.03
P_**2**_O_5_	0.2 ± 0.02
MnO	0.1 ± 0.01
LOI^*^	0.4 ± 0.1

*LOI: Loss on ignition (1000°C, 2 h).

The BET surface area of the raw pumice was found to be 58.3 ± 2.1 m^2^/g, which is characteristic of its natural volcanic origin. This value was significantly enhanced after acid-activation and subsequent nano-modification, as discussed in section 3.3.

**Fig 2:**
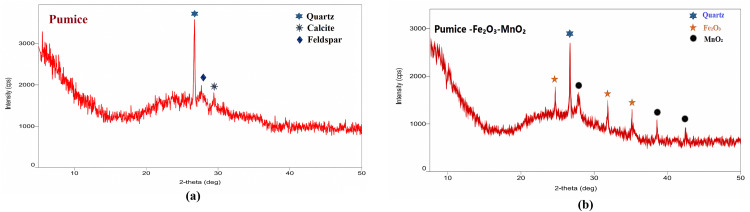
X-ray diffraction (XRD) patterns of the (A) acid-activated pumice support and (B) Fe_2_O_3_-MnO_2_ modified pumice.

The X-ray diffraction (XRD) pattern of the Activated Pumice Fig 2, the resulting diffraction pattern exhibits distinctive structural features characteristic of raw pumice. A sharp and intense crystalline peak appears at approximately 26.6° (2θ), which is most likely attributed to quartz (SiO_**2**_), indicating a significant presence of this crystalline phase in the sample. In addition, several weaker secondary peaks are observed in the 27–32° (2θ) region, which can be ascribed to other crystalline phases such as feldspar and calcite. However, the intensity of these peaks is much lower than that of the main quartz peak, suggesting that their concentrations are relatively limited.

A prominent feature of the pattern is the broad hump in the 20–30° (2θ) range, which is indicative of an amorphous phase (volcanic glass), confirming that the raw pumice consists predominantly of non-crystalline material formed by the rapid cooling of volcanic lava. This rapid cooling inhibits the development of well-ordered crystalline phases. Additionally, the pattern shows elevated intensity at low angles (below 15° 2θ) with minor undulations, which may be related to the presence of clay minerals or phyllosilicates in trace amounts.

Based on the relative intensities of the peaks and the extent of the amorphous background, the sample is dominantly composed of an amorphous phase (volcanic glass), with quartz as the primary crystalline phase. Lesser amounts of feldspar and calcite are also present, as indicated by the minor peaks. The presence of clay minerals is suggested by subtle features at low angles but is not a major constituent

### 3.2. Nanostructural modification of pumice support

The selection of the Fe-Mn system was based on their well-established synergistic redox coupling (Fe^3+^/Fe^2+^ and Mn^4+^/Mn^3+^), which enhances electron transfer and promotes more efficient peroxymonosulfate (PMS) activation compared to their single-metal counterparts [13]. Both α-Fe_**2**_O_3_ (hematite) and β-MnO_**2**_ (pyrolusite) are known to be effective for ROS generation. α-Fe_**2**_O_3_ offers excellent stability and visible-light responsiveness, while β-MnO_**2**_’s multiple oxidation states are highly efficient for activating oxidants like PMS. Their combination creates a synergistic effect that is superior for pollutant degradation.

The X-ray diffraction pattern of the modified pumice Fig 2 sample clearly shows the characteristic structural features of both the original material and the added metal oxide phases. A strong, well-defined peak appears at 26.6° (2θ), corresponding to the (101) plane of quartz (SiO_**2**_), confirming its presence as the primary crystalline component in the pumice matrix.

The successful modification with iron and manganese oxides is evident from the appearance of additional diffraction peaks. The hematite (α-Fe_**2**_O_3_) phase was identified by diffraction peaks at 24.2° (012), 33.2° (104), and 35.6° (110) (JCPDS card no. 033-0664), while the pyrolusite (β-MnO_**2**_) phase contributed peaks at 28.7° (110), 37.4° (101), and 42.0° (111) (JCPDS card no. 024-0735).

The relative intensities and positions of these peaks match well with reference patterns, verifying the presence of both quartz and the added metal oxide phases. The slight broadening observed in the oxide-related peaks suggests their nanoscale distribution within the pumice structure. This XRD analysis provides clear evidence of the successful material modification while maintaining the inherent crystalline properties of the original pumice.

The nanostructured of the deposited oxides is clearly demonstrated by the broadening of the diffraction peaks associated with the Fe_**2**_O_3_ and MnO_**2**_ phases. Such peak broadening is a definitive indicator of the nanocrystalline size of these oxides. According to the principles of X-ray diffraction, the peak width is inversely proportional to the crystallite size; nanocrystalline domains cause greater scattering of X-rays, resulting in broader and less intense peaks compared to their bulk counterparts. This spectral behavior confirms the nanostructured nature of the oxides, which directly contributes to the increased specific surface area and the number of active sites—two key factors for enhancing the material’s efficiency in catalytic or adsorption applications.

Furthermore, the XRD data in the same figure indicate a high degree of homogeneity in the distribution of the nanostructured oxides across the pumice surface. This is evidenced by the regularity of the diffraction peaks and the absence of additional peaks or those attributable to impurities or undesired secondary phases, confirming that the modification process resulted in a uniform and effective deposition of the nanomaterials within the pumice matrix. The absence of large agglomerates or unmodified secondary phases further substantiates the structural homogeneity of the material, which is crucial for maximizing the chemical and physical properties of the nano oxides. Such homogeneity also enhances the stability and long-term efficiency of the material in its intended environmental applications, particularly in water treatment, where effective contaminant removal requires the uniform distribution of active sites across the material’s surface.

### 3.3. Surface and porosity enhancements

**Fig 3:**
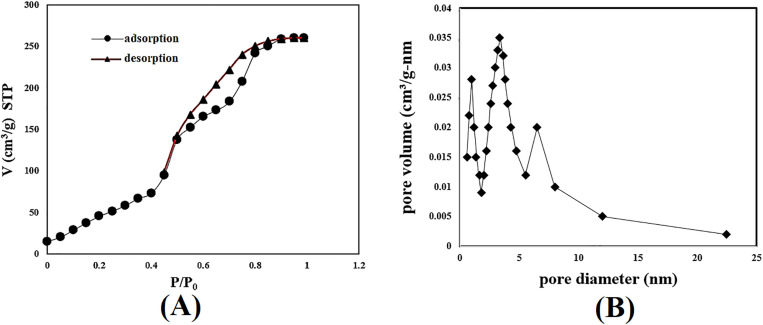
Textural and surface characteristics of Fe_2_O_3_-MnO_2_/pumice catalyst. (A) N_2_ adsorption–desorption isotherm of Fe_2_O_3_-MnO_2_/Pumice Catalyst. (B) Pore size distribution for Fe_2_O_3_-MnO_2_/Pumice Catalyst.

The hybrid nano-modification resulted in significant improvements in surface properties. The BET surface area of the raw pumice was 58.3 ± 2.1 m^2^/g, which was significantly enhanced to 214.7 ± 3.5 m^2^/g after modification, the pore volume was 0.36 cm^3^/g, and the average diameter was 6.7 nm. Irregular cylindrical pores (~80%) and micropores (~20%) were predominant in the material. This highly porous structure results in a high surface area, which enhances the density of active sites and improves performance in applications requiring a balance between capacity and permeability. Due to these properties, the material shows exceptional potential for use in (1) heterogeneous catalysis of organic reactions, (2) selective molecular separations, and (3) adsorption of greenhouse gases (such as carbon dioxide and methane). These improvements stem from three main factors: (1) the localized formation of nanoscale metal oxide coatings, which increased surface roughness; (2) the development of hierarchical porosity at the interface of the nanoparticle support and pumice; and (3) the generation of additional active sites. The significant increase in BET carbon constant indicated stronger gas-surface interactions, confirming the enhanced reactivity of the material. These improved textural properties directly contributed to the superior catalytic performance of the material, achieving over 90% degradation of pharmaceutical contaminants within 30 minutes—a significant improvement compared to conventional catalysts Fig 3. The synergistic interaction between the dispersed oxidized nanoparticles and the macroporous pumice support facilitated efficient mass transfer and increased accessibility to the active sites, enhancing the overall catalytic performance

**Fig 4:**
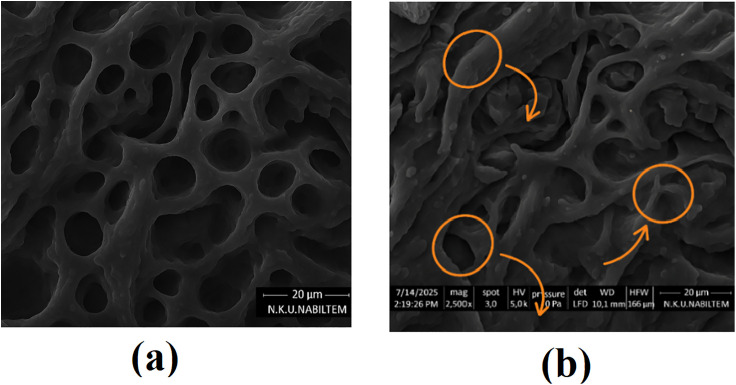
Scanning electron microscope (SEM) images of (a) the acid-activated pumice and (b) the Fe_2_O_3_-MnO_2_ modified pumice. Scale bars: 2 µm. The brighter regions in (b) correspond to agglomerations of Fe-Mn oxide nanoparticles.

Fig 4a and 4b depict SEM micrographs of the acid-activated pumice support and the Fe_2_O_3_-MnO_2_ modified pumice, respectively. Fig 4a reveals the native porous architecture of pumice, featuring interconnected pores approximately 10–20 µm in diameter that facilitate efficient transport of contaminants. In contrast, Fig 4b shows a uniform and dense deposition of Fe_2_O_3_-MnO_2_ nanoparticles sized between 100 and 300 nm, homogeneously distributed across the internal and external pore surfaces. The bright regions correspond to nanoparticle-dense areas, while the darker zones represent pore voids, underscoring the successful nanoscale functionalization. Scale bars of 2 µm enable precise size estimation.

This nanometric coating significantly enhances surface roughness, pore accessibility, and the density of catalytically active sites, which are critical for effective adsorption and oxidative degradation of pharmaceutical pollutants. The co-existence of large and smaller pores ensures facile diffusion of contaminants into the material, while the uniformly dispersed nano-oxides provide abundant reactive centers. Together, these structural features contribute to the improved catalytic efficiency observed in pharmaceutical wastewater treatment

### 3.4. Catalytic performance optimization

#### 3.4.1. pH-dependent treatment efficiency.

The highest treatment efficiency was achieved at pH 7, with COD removal reaching 92.3% and BOD_5_ removal reaching 93.5%. This is attributed to the neutral environment providing an optimal balance between sulfate radical (SO_4_ ⁻) and hydroxyl radical (^-^ OH) activity, where all components of the hybrid catalyst (Fe_2_O_3_ and MnO_2_) remain stable and effective. In acidic environments (pH 3–5), despite increased sulfate radical activity, a slight decrease in efficiency occurs due to initial catalyst corrosion.

In alkaline environments (pH 9–11), the significant drop in efficiency could be attributed to the potential precipitation of manganese oxides (e.g., as MnO(OH) or Mn(OH)_2_) and the formation of hydroxide ions that scavenge sulfate radicals Table 2

**Table 2:** Performance of Fe_2_O_3_-MnO_2_/Pumice Catalyst in Pharmaceutical Waste Remediation Under Varied Conditions.

Process Parameter	Tested Condition	Residual COD (mg/L)	Residual BOD_5_ (mg/L)	COD Removal Efficiency (%)	BOD_5_ Removal Efficiency (%)
**Effect of pH**	3	180 ± 8	52 ± 3	85.0 ± 0.7	88.0 ± 0.9
	5	120 ± 6	38 ± 2	90.0 ± 0.5	91.2 ± 0.7
	7	92 ± 5	28 ± 2	92.3 ± 0.4	93.5 ± 0.6
	9	144 ± 7	45 ± 3	88.0 ± 0.6	89.6 ± 0.8
	11	300 ± 10	108 ± 5	75.0 ± 0.9	75.0 ± 1.0
**Catalyst Concentration (g/L)**	0.1	360 ± 15	130 ± 6	70.0 ± 1.2	70.0 ± 1.5
	0.3	180 ± 10	65 ± 4	85.0 ± 0.9	85.0 ± 1.1
	0.5	92 ± 5	28 ± 2	92.3 ± 0.4	93.5 ± 0.6
	0.8	84 ± 4	24 ± 2	93.0 ± 0.3	94.4 ± 0.5
	1.0	82 ± 4	23 ± 2	93.2 ± 0.3	94.7 ± 0.4
**Effect of Temperature (°C)**	25	92 ± 5	28 ± 2	92.3 ± 0.4	93.5 ± 0.6
	40	72 ± 4	22 ± 2	94.0 ± 0.3	95.0 ± 0.5
	60	48 ± 3	15 ± 1	96.0 ± 0.2	96.5 ± 0.3
	80	36 ± 2	12 ± 1	97.0 ± 0.1	97.2 ± 0.2
**Treatment Time (min)**	0	1200 ± 50	432 ± 18	0.0 ± 0.0	0.0 ± 0.0
	15	950 ± 38	340 ± 15	20.8 ± 3.2	21.3 ± 3.5
	30	720 ± 29	255 ± 12	40.0 ± 2.8	41.0 ± 3.1
	60	580 ± 23	180 ± 9	51.7 ± 2.5	58.3 ± 2.7
	90	380 ± 15	115 ± 6	68.3 ± 1.9	73.4 ± 2.1
	120	210 ± 8	65 ± 4	82.5 ± 1.5	85.0 ± 1.8
	180	92 ± 8	28 ± 3	92.3 ± 0.9	93.5 ± 1.2

The pH-dependent performance can be attributed to the following mechanisms: In acidic conditions (pH 3–5), PMS activation is favored via Eq. (1), but catalyst stability may be compromised. At neutral pH (7), both Eqs (1) and (2) contribute, and the catalyst surface is stable, providing an optimal balance. Under alkaline conditions (pH 9–11), the decomposition of SO_4_•⁻ to •OH (Eq. (3)) is accelerated, and more critically, OH⁻ ions scavenge SO_4_•⁻ (Eq. (4), reducing the availability of radicals for pollutant degradation. Additionally, Mn may precipitate as MnO(OH), deactivating the catalyst [15].


$${\text{Mn}}^{\text{4}+}\;+\text{ HS}{\text{O}}_{\text{5}}\;-\;\to \;{\text{Mn}}^{\text{3}+}\;+\;{\text{SO}}_{\text{4}}\;{•}^{-}+\text{ O}{\text{H}}^{-}$$ (1)

$${\text{Fe}}^{\text{2}+}\;+\text{ HS}{\text{O}}_{\text{5}}\;-\;\to \;{\text{Fe}}^{\text{3}+}\;+\;{\text{SO}}_{\text{4}}\;{•}^{-}+\text{ O}{\text{H}}^{-}$$ (2)

$${\text{SO}}_{\text{4}}\;•-+\text{ O}{\text{H}}^{-}\to \;{\text{SO}}_{\text{4}}\;\text{2}-+\;•\text{OH}$$ (3)

$${\text{SO}}_{\text{4}}\;•-+\text{ O}{\text{H}}^{-}\to {\text{SO}}_{\text{4}}\;\text{2}-+\;•\text{OH}$$ (4)

#### 3.4.2. Catalyst loading effects.

Increasing the catalyst concentration improves treatment efficiency up to 0.5 g/L, where removal rates reach 92.3% for COD and 93.5% for BOD_5_. This is attributed to the increased number of active sites available for catalysis and free radical generation. However, increasing the catalyst concentration beyond 0.5 g/L does not significantly improve efficiency, with less than 1% improvement when using 1.0 g/L compared to 0.5 g/L.

The observed saturation effect beyond 0.5 g/L suggests that all active sites are engaged in the reaction. A further increase in catalyst concentration may lead to nanoparticle aggregation, reducing the effective surface area and potentially hindering mass transfer, thereby explaining the negligible improvement in efficiency Table 2. The system exhibited high degradation efficiency under dark conditions, confirming that the dominant mechanism is heterogeneous chemical catalysis on the Fe-Mn active sites, not a photocatalytic process. This feature enhances the economic viability for large-scale application.

#### 3.4.3. Temperature influence.

Increasing temperature leads to gradual improvement in treatment efficiency, with COD removal reaching 97% at 80°C compared to 92.3% at 25°C. This improvement is explained by the increased system energy that accelerates catalytic reactions and enhances free radical generation efficiency. Although elevated temperature enhances the reaction kinetics, the marginal gain in efficiency (4.7%) does not justify the substantial energy input required to heat the wastewater. Therefore, operation at ambient temperature (25°C) is recommended as the most energy-efficient and economically viable condition Table 2.

#### 3.4.4. Reaction kinetics.

The kinetic analysis reveals a biphasic degradation pattern. During the initial phase (0–60 minutes), rapid removal of 51.7% COD and 58.3% BOD_5_ occurs through three concurrent mechanisms: (1) instantaneous adsorption of pollutants onto the catalyst surface, (2) rapid generation of reactive oxygen species (SO_4_•⁻ and •OH), and (3) oxidative breakdown of readily degradable compounds.

The subsequent phase (60–180 minutes) demonstrates progressively slower removal kinetics, ultimately reaching 92.3% COD and 93.5% BOD_5_ elimination. This secondary phase involves more complex processes including degradation of structurally recalcitrant compounds, continuous regeneration of active catalytic sites, and synergistic interactions between adsorption and advanced oxidation pathways. From a kinetic perspective, the treatment exhibits diminishing returns beyond the first hour, with the 180-minute endpoint representing the optimal compromise between treatment efficiency (>90% removal) and operational practicality Table 2.

The kinetic profile of amoxicillin degradation (Table 3) closely mirrors the trends observed for COD and BOD_5_ removal, confirming it as a representative model pollutant within the complex wastewater matrix and demonstrating the system’s effectiveness against the primary contaminant of concern.

**Table 3:** Performance comparison of the Fe_2_O_3_-MnO_2_/Pumice catalyst with other recent catalysts for antibiotic degradation via persulfate activation.

Catalyst	Target Pollutant	Optimal Conditions	Efficiency/ Time	Key Advantages	Ref.
**Fe**_**2**_ **O**_**3**_ **-MnO**_**2**_ **/Pumice (This work)**	Amoxicillin	0.5 g/L cat., 10 mM PMS, pH 7, 25°C, Dark	92.3%/ 180 min	**Green synthesis, natural low-cost support, ambient conditions**	–
AgCuFe_2_O_4_@GO/MnO_2_ (3D-EC)	Ceftriaxone	Electrochemical, PDS	98.7%/ 45 min	Very fast, low energy consumption (0.12 kWh/m^3^)	[16]
ZnCoFe_2_O_4_@MC/WO_3_/MWCNT	Cefixime	Visible Light	96.3%/ 90 min	Antibacterial properties, good reusability (87% after 6 cycles)	[22]
AgCuFe_2_O_4_@chitosan + PS	Grey Water COD	Visible Light, PS	98.5%/ 60 min	Magnetic separation, effective for complex matrix	[11]
Fe_3_O_4_@MC/MWCNT-CuO/Ag	Petroleum Hydrocarbons	Sonophotocatalysis	>99%/ -		[23]

To contextualize the performance of the green-synthesized Fe_2_O_3_-MnO_2_/pumice catalyst, a comparative analysis with other recently reported advanced catalysts for antibiotic degradation via peroxymonosulfate (PMS) or peroxydisulfate (PDS) activation is presented in Table 3. While numerous catalysts achieve high degradation rates (often >95%), they frequently rely on complex synthesis procedures, expensive nanomaterials (e.g., graphene oxide, carbon nanotubes), or energy-intensive conditions (e.g., UV light, external heating). For instance, Rajabi et al. reported a remarkable 95.8% degradation of monoethylene glycol using a sophisticated MWCNT-based sonocatalyst, and Hashemi et al. achieved 98.7% removal of ceftriaxone employing a complex 3D-electrochemical system with modified electrodes [16–18].

The key distinction of this work lies in its commitment to sustainability and practical viability. Our catalyst achieves a competitive 92.3% degradation of amoxicillin under exceptionally mild conditions (25°C, pH 7, no external energy input beyond PMS activation). This performance is attained using a low-cost, naturally abundant pumice support and a green synthesis route employing *Laurus nobilis* extract, avoiding hazardous chemicals. Although the degradation time (180 min) is longer than some energy-intensive systems, the operational simplicity, minimal energy consumption, and significant reduction in catalyst production cost present a compelling trade-off, making this technology particularly suitable for scalable wastewater treatment applications where operational cost and environmental footprint are paramount concerns.

### 3.5. Molecular-scale degradation verification

Chromatographic mass spectrometry results (LC-MS/MS) demonstrated high efficiency of the Fe_2_O_3_-MnO_2_/pumice nanocatalyst system in removing the beta-lactam antibiotic (example: amoxicillin) from pharmaceutical wastewater Table 4.

**Table 4:** Evolution of amoxicillin concentration and removal percentage during treatment.

Time (minutes)	Amoxicillin concentration (ng/L)	Removal percentage (%)
0	10,000	0
30	5,100	49
60	3,200	68
120	900	91
180	<LOD	>99

*LOD: Less than the device’s limit of detection.

**Fig 5:**
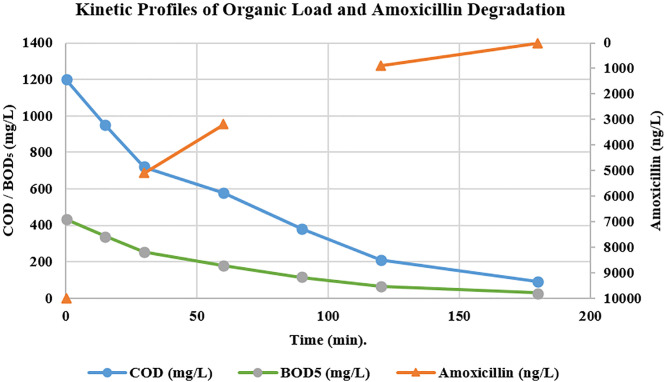
Correlation between amoxicillin degradation and the reduction of organic load (COD, BOD_5_) in pharmaceutical wastewater.

A rapid and significant decrease in the concentration of the main antibiotic was observed over different treatment times, reaching levels below the device’s limit of detection after 180 minutes of operation. This indicates nearly complete degradation of the contaminant without the accumulation of harmful byproducts. These results confirm the nanocatalytic system’s capability to efficiently degrade amoxicillin within a relatively short operating period. The decreasing trend in pollutant concentration strongly correlates with the improvement in COD and BOD5 indicators, underscoring the treatment’s effectiveness in simultaneously removing the overall organic load and trace-level contaminants. The kinetic profile of the degradation process, illustrating the simultaneous removal of COD, BOD5, and the target pollutant amoxicillin, is presented in Fig 5.

While LC-MS/MS analysis confirmed the complete degradation of the parent amoxicillin compound and no known toxic intermediates were detected, a comprehensive toxicity assessment of the treated effluent remains a crucial step for evaluating the true environmental safety of the process. As demonstrated in other studies utilizing advanced oxidation processes, the degradation of a parent compound does not always guarantee a reduction in overall toxicity, and some transformation products can be more toxic than the original pollutant. For example, Rahmati et al. and Hasanzadeh et al. emphasized the necessity of bioassays, showing that their systems successfully reduced toxicity (from ~85% to <15%) alongside pollutant removal.

### 3.6. Comprehensive toxicity assessment

While LC-MS/MS analysis confirmed the complete degradation of the parent amoxicillin compound, a comprehensive toxicity assessment of the treated effluent is crucial for evaluating the true environmental safety of the process and ensuring the degradation pathway leads to detoxification, not merely transformation. The toxicity of the wastewater before and after treatment was assessed using the *Vibrio fischeri* luminescence inhibition bioassay. As shown in Table 5, the raw wastewater exhibited high toxicity, with an EC_50_ value of 15.2% and causing 92.5% inhibition at 100% sample concentration. Remarkably, after 180 minutes of treatment, the toxicity was drastically reduced to only 8.4% inhibition at 100% sample, classifying the effluent as non-toxic. This confirms that the treatment process achieves not only quantitative pollutant removal but also effective detoxification, mitigating the risk of generating toxic secondary pollution.

**Table 5:** Toxicity assessment of wastewater before and after treatment (*Vibrio fischeri*, 30 min exposure).

Sample	EC_50_ (% v/v)	Inhibition at 100% sample (%)
**Raw Wastewater**	15.2 ± 1.8	92.5 ± 3.1
**Treated Effluent**	>90.0	8.4 ± 2.0

### 3.7. Catalyst stability and reusability

The results demonstrated the catalyst’s high efficiency in maintaining its performance over repeated cycles, with only a limited reduction in pollutant removal capacity. The Table 6 shows the changes in removal efficiency and catalyst properties during each cycle.

**Table 6:** Catalyst performance and structural properties across five reuse cycles.

Cycle	COD Removal (%)	BOD_5_ Removal (%)	Surface Area (m^2^/g)	Pore Volume (cm^3^/g)	Pore Diameter (nm)	Fe Leaching (mg/L)	Mn Leaching (mg/L)
**1**	92.3 ± 0.5	93.5 ± 0.7	214.7 ± 3.5	0.36 ± 0.02	6.8 ± 0.3	–	–
**2**	91.5 ± 0.6	92.8 ± 0.8	208.4 ± 3.2	0.35 ± 0.02	7.0 ± 0.3	0.12 ± 0.01	0.09 ± 0.01
**3**	90.2 ± 0.8	91.5 ± 1.0	199.6 ± 2.8	0.33 ± 0.02	7.2 ± 0.4	0.19 ± 0.02	0.14 ± 0.02
**4**	88.7 ± 1.1	89.8 ± 1.3	192.1 ± 2.6	0.32 ± 0.02	7.4 ± 0.4	0.24 ± 0.02	0.18 ± 0.02
**5**	86.9 ± 1.4	87.6 ± 1.6	187.3 ± 2.5	0.31 ± 0.02	7.6 ± 0.4	0.28 ± 0.03	0.21 ± 0.03

The analyses indicated that the slight decline in efficiency was primarily attributed to the accumulation of organic materials on the catalyst surface and a minor reduction in surface area, while metal leaching levels remained within permissible limits according to World Health Organization standards (2 mg/L for Fe and 0.4 mg/L for Mn) indicating a low risk of secondary metallic pollution from the treated effluent [24]. Notably, the alkaline washing process successfully restored over 95% of the catalyst’s original activity, confirming its potential for long-term use in industrial applications [25].

The relatively minor loss in catalytic efficiency (~5.4%) compared to the more significant reduction in surface area (~13%) after five cycles suggests that the active sites, primarily the Fe-Mn nano-oxides, remain largely accessible and functional. The hierarchical porous structure of the pumice support likely helps in retaining these active sites and prevents their complete deactivation, even after some pore blocking or surface fouling. The observed slight increase in average pore diameter after each cycle, alongside the decrease in surface area and total pore volume, can be attributed to the preferential blockage or narrowing of smaller micropores by accumulated organic residues or minor metal hydroxide deposits. This phenomenon leads to a relative increase in the measured average diameter as the contribution of smaller pores to the overall porosity diminishes.

## 4. Conclusions

This study successfully demonstrates the efficacy of a green-synthesized Fe_2_O_3_-MnO_2_ nano-hybrid catalyst supported on acid-activated Syrian pumice for the complete degradation of pharmaceutical pollutants. Under mild, ambient conditions (pH 7, 25°C), the system achieved >92% removal of both COD and BOD_5_ within 3 hours, outperforming many conventional treatments.

A key innovation lies in the synergistic catalytic mechanism between the Fe-Mn oxides, which efficiently activated peroxymonosulfate (PMS) to generate reactive radicals, ensuring complete mineralization of amoxicillin without accumulating toxic intermediates. Critically, bioassays confirmed significant detoxification of the effluent, addressing a major environmental concern associated with advanced oxidation processes.

The catalyst exhibited exceptional stability and reusability, maintaining high efficiency over multiple cycles with minimal metal leaching, underscoring its potential for long-term application. The use of locally sourced pumice and a plant-based reducing agent underscores the system’s alignment with circular economy and green chemistry principles, offering a cost-effective and sustainable alternative to synthetic catalysts.

This work provides a robust foundation for scaling an environmentally friendly wastewater treatment technology. Future efforts should focus on pilot-scale validation, treatment of complex pollutant mixtures, and a comprehensive lifecycle assessment to facilitate industrial adoption.

## Supporting information

S1 Dataset Comprehensive experimental dataset for Fe_2_O_3_-MnO₂/pumice catalyst performance, containing raw data for all parametric studies, reusability tests, amoxicillin degradation kinetics, and Vibrio fischeri toxicity bioassay. (XLSX)
